# Organizational Climate and Decision Aid Sustainability in Lupus Care: Mixed Methods Study

**DOI:** 10.2196/69603

**Published:** 2025-08-21

**Authors:** Aizhan Karabukayeva, Larry R Hearld, Nathan W Carroll, Reena Kelly, Jasvinder A Singh

**Affiliations:** 1 Hudson College of Public Health, University of Oklahoma Health Sciences Center, 801 NE 13th St., Oklahoma City, OK, 73014, United States, 1 2055155146; 2 Department of Health Services Administration, School of Health Professions, University of Alabama at Birmingham, Birmingham, AL, United States; 3 Department of Health Administration, College of Health Professions, Virginia Commonwealth University, Richmond, VA, United States; 4 Department of Population Health and Leadership, School of Health Sciences, University of New Haven, West Haven, CT, United States; 5 Section of Immunology, Allergy and Rheumatology, Baylor College of Medicine, Houston, TX, United States

**Keywords:** organizational climate, organizational readiness for change, organizational learning, lupus, decision-aid, sustainment

## Abstract

**Background:**

Digital decision aids (DAs) are increasingly used in health care to support shared decision-making and promote patient engagement. In the context of systemic lupus erythematosus (SLE), a complex autoimmune disease characterized by diverse symptoms and uncertain prognoses, DAs offer guidance to patients in navigating treatment risks and benefits. Although numerous studies have examined the initial implementation of evidence-based tools, there is limited evidence on the organizational factors that influence their long-term sustainability in clinical practice. This gap is particularly salient for digital interventions, where integration into routine workflows and ongoing use require alignment with clinic readiness and culture. This study focuses on an evidence-based, electronic DA designed to support patients with lupus and investigates how dimensions of organizational climate, particularly learning climate and change readiness, are associated with the tool’s sustained use across diverse practice settings.

**Objective:**

This study aims to examine the relationship among the learning climate, change readiness climate, and perceived permanence of a DA for patients with lupus in 15 geographically diverse rheumatology clinics in the United States.

**Methods:**

This study was conducted as part of a broader multisite implementation project. We used a concurrent mixed methods design, integrating longitudinal quantitative survey data with qualitative interviews. Quantitative data were collected via web-based surveys at 3 time points (6-, 12-, and 24-mo postimplementation) from physicians, nurses, medical assistants, and administrative personnel (n=204 responses across rounds). The primary outcome was perceived DA permanence, measured with a validated 5-item scale. Independent variables included internal and external learning climates, change commitment, and change efficacy. Data were aggregated to the clinic level and analyzed using generalized estimating equations with clustered SEs. Qualitative data were collected through 36 semistructured interviews with clinic staff to explore contextual factors affecting sustainment.

**Results:**

Quantitative findings revealed that change efficacy climate was significantly associated with greater perceived permanence of the DA (β=4.00; *P*<.001) while internal (β=−1.39; *P*<.05) and external learning climates (β=−2.11; *P*<.01) were negatively associated. Change in commitment was not statistically significant. Qualitative data highlighted challenges to sustainment, including poor workflow integration, lack of physician buy-in, and limited applicability of the DA to certain patient populations.

**Conclusions:**

Sustaining digital health tools like DAs requires not only technical integration but also a supportive organizational climate. This study demonstrates that perceptions of a clinic’s collective ability to sustain change (change efficacy) are critical while learning climates may expose barriers that hinder long-term use. These findings underscore the importance of assessing organizational readiness and tailoring implementation strategies to foster DA sustainment in real-world settings.

## Introduction

As health care increasingly embraces digital innovation, electronic decision aids (DAs) have emerged as vital tools for enhancing shared decision-making, improving patient engagement, and promoting patient-centered care [1,2]. These tools deliver evidence-based information about treatment options in accessible formats, empowering patients to make informed decisions aligned with their values and preferences [3,4]. In chronic and complex conditions, such as systemic lupus erythematosus (SLE), digital DAs can be especially impactful by helping patients understand long-term treatment implications and participate more meaningfully in their care [5], potentially improving adherence to complex medication regimens, a known challenge in SLE management [6].

Lupus is a chronic autoimmune condition characterized by a relapsing-remitting course and variable clinical presentations, ranging from mild symptoms to severe, life-threatening complications [7]. Characterized by disease unpredictability, complex medication regimens, and significant quality-of-life impacts [8], lupus often requires nuanced treatment decisions—particularly for women of childbearing age or patients with organ involvement [9,10]. However, time constraints in outpatient settings [11], variable health literacy [12], and limited consultation windows can inhibit the level of discussion needed for truly informed choices. Digital DAs offer a scalable, patient-centered solution to these challenges by preparing patients in advance of clinical encounters, encouraging questions, and fostering dialogue during visits [13,14], providing information at the patient’s own pace, and facilitating structured discussions.

Despite their promise, many digital health tools, including DAs, are not sustained in routine care beyond their initial implementation phase [15]. Research indicates that adoption alone is not sufficient. Digital interventions must be actively maintained and integrated into clinical workflows to generate lasting impact [16]. Challenges to sustain often stem from a lack of organizational readiness, weak leadership support, poor workflow fit, and minimal clinician engagement [17-19]. While implementation science has extensively examined adoption and fidelity, the long-term sustainability of DAs in real-world clinical environments remains an understudied area—especially as it relates to digital health innovation [20].

Organizational climate—the shared perceptions of policies, practices, and procedures—plays a central role in shaping implementation success and long-term sustainment [17,21]. Two specific climate dimensions may be especially relevant for sustaining DAs: learning climate and readiness for change. A positive internal learning climate, characterized by team-level reflection and feedback, and an external learning climate, characterized by openness to outside knowledge and innovation, are theorized to foster the integration of digital tools into daily workflows (Edmondson [22]). Therefore, we hypothesize that a more positive internal learning climate (Hypothesis 1a; H1a) and external learning climate (Hypothesis 1b; H1b) will each be associated with greater perceived permanence of the DA.

In parallel, organizational readiness for change, defined as the collective willingness (change commitment) and confidence (change efficacy) to implement and sustain new practice, is also expected to support long-term use of innovations (Weiner [17]; Shea et al [23] 2014). Clinics with stronger change commitment may be more motivated to continue using DA, while higher change efficacy may promote creative problem-solving and proactive integration. Thus, we hypothesize that a more supportive climate of change commitment (H2) and change efficacy (H3) will be positively associated with the perceived permanence of DA in routine care. To test these hypotheses, we examined data from a multisite implementation of a digital lupus DA across 15 geographically diverse rheumatology clinics in the United States. Our goal was to identify key organizational factors associated with the long-term integration and sustained use of the DA, with particular attention to how learning climate and readiness for change contribute to its long-term integration.

## Methods

### Study Design and Setting

A concurrent mixed methods approach was used for the study. Quantitative data identified patterns across large numbers of study participants and sites while qualitative data were used to examine the underlying reasons for these patterns by understanding clinic personnel’s perspectives on the perceived permanence of the DA [24]. Each data strand was collected and analyzed independently, followed by integration through triangulation to develop a comprehensive picture of DA sustainment. The study was part of a larger evaluation of the effectiveness of different implementation strategies for helping 15 clinics throughout the United States implement and sustain the use of a culturally competent, electronic DA designed to educate patients with lupus about their treatment options and help them engage in more shared decision-making with their clinical care team. The detailed description of the DA for patients with lupus is described elsewhere [25].

### Data Sources

#### Quantitative Survey Data

The study relied primarily on 2 quantitative data sources. The first data source was a baseline web-based survey of members of the lupus clinics, including physicians, nurses, clinic administrators, medical assistants, front desk personnel, and research study coordinators. This “organizational assessment” survey included organizational climate measures (organizational readiness for change and learning), as well as clinic-level characteristics (eg, clinic size and ownership). A sampling frame was constructed for each site by asking the clinic study coordinator at each of the 15 clinics to provide the names and email addresses of all clinic personnel who would be working with or affected by the DA of patients with lupus. An email was then sent to each person in the sampling frame informing them about the purpose of the study and the survey, an estimate of the time required to complete the survey (10 min), and a hyperlink to the survey. Reminder emails were sent to nonrespondents 3 and 7 business days after the initial email.

The second data source was a web-based “implementation” survey administered at 3 different times over the study period: Round 1 was administered 6 months after the DA was first implemented in the clinic; Round 2 was administered 1 year after the DA was implemented in the clinic, and Round 3 was administered 2 years after the DA was implemented in each clinic. The main objective of this survey was to assess the clinic personnel’s perception of the permanence of the DA. The sampling frame for the survey consisted of all clinic members who either directly interacted with the DA (eg, study coordinators) or were affected by its use (eg, physicians, nurses, medical assistants, and clinic administrators).

#### Qualitative Interview Data

The third data source was semistructured interviews with the clinic personnel to provide more in-depth understanding of the opportunities for sustaining the DA. Data collection took place during May-July 2022 via a cloud-based video conferencing service (Zoom; Zoom Communications). The average duration of the interviews was 20 (SD 10; range 10‐30) minutes. Either the study coordinator, the principal investigator, or both at each site clinic selected key informants from a variety of positions within a clinic to assure that a wide variety of perspectives on potential challenges to sustaining the DA was captured. The eligibility criteria included being an employee of the clinic and being familiar with the clinic’s research and patient care activities. Three research team members (LH, AK, and RK) piloted the initial interview protocol with 3 key informants to assess the clarity of questions, identify gaps in the protocol, and improve the flow of the interview. Subsequent interviews were conducted by these same 3 research team members.

**Figure 1.**
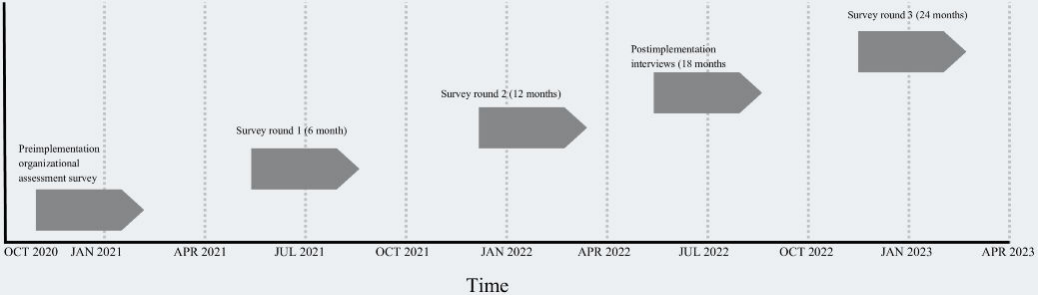
Implementing the Decision-Aid for Lupus study timeline: Data collection phase.

The interview protocol for clinic personnel included questions related to the overall perceptions and experiences with the DA and views regarding its long-term sustainment (Multimedia Appendix 1). Participants were asked about their general involvement with the project, probing specifically for positive and negative experiences with the DA that might impact the sustainment. In addition, participants were asked to provide more targeted opinions on organizational factors that affect continued use of the DA, including the type of support and resources needed to effectively sustain the DA. The interview protocol for clinic personnel was constructed based on the patient experience with DAs [1]. Figure 1 provides an overview of the timing and duration of each data collection activity across the study period, including the preimplementation assessment, implementation-phase surveys, and postimplementation interviews.

### Measures

#### Outcome Variable

Perceived DA permanence of the DA, the primary outcome, was assessed via implementation survey at 6, 12, and 24 months postimplementation. Perceived DA permanence was measured using 5 validated items that were scored ranging from 1 (“Disagree”) to 5 (“Agree”) [26]. The 5 items were averaged to construct a composite score reflecting perceived DA permanence, where higher scores indicate perceptions of greater permanence of the DA in the clinic (ie, better outcome).

#### Predictor Variables

The primary predictor variables examined in this study were 4 summated scales based on multiple Likert-type items from the web-based survey (Multimedia Appendix 2).

There is psychometric evidence in support of a measure of organizational readiness for change, called Organizational Readiness for Implementing Change (ORIC) [23]. It is a brief, reliable, and valid measure that differentiates between change commitment and change efficacy. Commitment was operationalized as the average of 5 survey items, all of which were measured on a 5-point scale ranging from 1=Disagree to 5=Agree. Similarly, change efficacy was operationalized as the average of 7 survey items measured on the same 5-point scale.

The organizational learning variable was measured using the Team Learning Survey developed by Edmondson [27]. Organizational learning was divided into 2 dimensions, such as internal and external learning. Internal learning climate was an average of 8 survey items, and external learning climate was an average of 4 survey items, all items measured on a 7-point scale (1=Strongly disagree and 7=Strongly agree).

#### Covariates

Additional clinic-level variables included 5 clinic characteristics that reflected structural, cultural, and general staffing attributes of the clinic. Clinic specialty was a dichotomous variable that indicated whether a clinic included only a single medical specialty (ie, rheumatology; coded 0) or multiple medical specialties (coded 1). Ownership was also a dichotomous variable that indicated whether a clinic was owned by a university/part of an academic medical center (coded 0) or not owned by a university (coded 1). Clinic size was operationalized as a series of dummy variables reflecting 3 categories: 1‐10 members (referent); 11‐30 members; and more than 30 members. The analysis included a count variable to account for the number of physical clinic locations for each participating site. Individual years of experience were averaged at the clinic level to create a continuous variable that reflected the work experience of clinic members. Finally, clinic culture was based on responses to 4 survey items that asked respondents to allocate percentage points (0%‐100%) to four different culture types [28,29]: (1) Clan/Team culture (friendly workplace where leaders act like mentors/facilitators and there is an emphasis on doing things together); (2) Rational/Hierarchical culture (structured and formalized workplace where leaders act like coordinators and value is placed on doing things right); (3) Adhocracy/Entrepreneurial culture (dynamic workplace with leaders that stimulate new ideas and value is placed on doing things first); and (4) Market culture (competitive workplace with leaders that are like producers and value placed on doing things fast). Individual responses were aggregated to the clinic level by averaging across clinic members. A clinic was then assigned a dominant culture type (eg, clan/team culture) if, on average, respondents assigned greater than 50% points to that culture type. For clinics where responses, on average, did not reveal a dominant culture type (ie, were more evenly distributed across the 4 culture types), they were labeled as having no dominant culture (referent group).

#### Quantitative Analytic Strategy

Organizational climate measures were aggregated to the clinic level. To justify aggregating individual responses to the clinic level, we calculated within-unit agreement and sufficient between-unit differences using 3 multi-item interrater agreement statistics: (1) Intraclass correlation coefficient; (2) Within group reliability (rwg(j)); and (3) Average Deviation (Adm(j)). Together, these analyses provided evidence that sufficient agreement existed among members’ perception of climate measurements to support aggregation to the clinic level (Table 1).

**Table 1.** Characteristics of survey respondents by implementation round of the DA permanence survey.

Respondent characteristics	Round 1 (N=77)	Round 2 (N=60)	Round 3 (N=70)
Response rate (%)	56.8	46.5	54.8
Age, n (%)			
19-24	9 (12)	4 (6)	5 (7)
25-44	38 (49)	31 (50)	32 (47)
45-64	26 (33)	24 (39)	28 (42)
More than 65	4 (5)	3 (4)	2 (2)
Sex, n (%)			
Male	19 (25)	17 (28)	25 (38)
Female	58 (75)	44 (72)	41 (62)
Race, n (%)			
White	43 (56)	36 (60)	39 (61)
Black or African American	13 (17)	4 (7)	7 (11)
Asian	21 (27)	20 (33)	18 (28)
Ethnicity, n (%)			
Hispanic or Latino	5 (7)	6 (10)	6 (9)
Not Hispanic or Latino	70 (93)	54 (90)	58 (91)
Education, n (%)			
High school graduate	1 (1)	1 (2)	2 (3)
Undergraduate or less	35 (46)	22 (35)	23 (34)
Professional degree	17 (22)	16 (26)	18 (27)
Doctorate	24 (31)	23 (37)	24 (36)
Role in the clinic, n (%)			
Physician	28 (37)	29 (48)	26 (39)
Other clinical personnel	22 (29)	11 (18)	20 (30)
Administrative personnel	26 (34)	21 (34)	21 (31)

The multivariate analysis used generalized estimating equations and clustered SEs at the clinic level to account for potential correlations of individual respondents within the same clinic. All predictor variables were mean-centered to facilitate model interpretation [30]. All analyses were performed using Stata version 17 (StataCorp LLC) [31] and all relationships were considered statistically significant at *P*<.05.

#### Qualitative Analytic Strategy

We conducted a thematic analysis guided by Braun and Clarke’s [32] 6-phase approach, which emphasizes reflexive and transparent practices in qualitative research. Each semistructured interview was recorded and transcribed verbatim. We used a hybrid inductive-deductive coding strategy where initial codes were drawn from the interview protocol (deductive) while additional themes were identified from the data (inductive) through iterative reading and comparison with literature on organizational change and sustainment. To ensure credibility and confirmability, 2 researchers (LH and AK) independently coded an initial subset of transcripts (n=3‐4), achieving strong interrater reliability (k>0.81). Discrepancies were discussed and resolved through consensus, after which the remaining transcripts were coded by AK and RK. NVivo 12 Plus (QSR International) software was used to facilitate coding and organize themes. Reflexivity was maintained through memo writing and analytic journaling at the clinic level, supporting dependability and transferability by documenting analytic decisions and contextual factors. Themes were developed through familiarization, coding, theme generation, review, and refinement, with representative quotes used to support interpretations.

#### Triangulation of Qualitative and Quantitative Data Sources

Quantitative and qualitative data were integrated using a convergent mixed methods design, with findings triangulated to provide a comprehensive understanding of sustainment. Two strands were linked through a joint display table that aligned survey results and interview insights by thematic categories derived from the research questions. This allowed for direct comparison and contextualization of quantitative findings, for example, scores on organizational climate domains, with qualitative narratives that explained the underlying processes. Interviews added depth by uncovering contextual factors and “behind-the-scenes” mechanisms influencing continued use of the DA. Themes emerging from this integration informed the interpretation and organization of study implications.

### Ethical Considerations

The study was approved by the Institutional Review Board (IRB) at the University of Alabama at Birmingham (UAB, 300002272; UAB Coordinating Center 200002554). Each study site’s IRB (15 sites including UAB) also reviewed and approved the study. The IRB approved the use of verbal informed consent given that the interviews were conducted via Zoom. During the consent process, clinical personnel were informed that participation was voluntary, and they could withdraw from the study at any time. All investigations were conducted in conformity with ethical principles of research. All study procedures adhered to the ethical principles outlined in the Belmont Report and complied with the Health Insurance Portability and Accountability Act (HIPAA). All study data were deidentified and stored securely on IRB-approved services, accessible only to authorized personnel. Each study participant was paid US $50 for completing the baseline survey assessment and US $10 for follow-up assessments.

## Results

### Quantitative Results

#### Respondent Characteristics

The overall response rate for the DA permanence survey was as follows: 56.8% for the Round 1 survey; 46.5% for the Round 2 survey; and 48.5% for the Round 3 survey across the 15 clinics. The demographic characteristics of the respondents are described in Tables2  3. In Round 1, 77 respondents completed the survey, and 58 (75%) respondents were female; 43 (56%) respondents were White, and 38 (49%) respondents were in the age group of 25‐44 years. Most respondents had an undergraduate degree (35, 45%) and around 28 (37%) respondents were physicians while 26 (34%) respondents were administrative personnel. In Round 2, there were 60 respondents, and in Round 3, there were 70 respondents. The demographic profile of the later rounds was similar to the respondents of Round 1.

**Table 2.** Baseline organizational climate characteristics from the organizational assessment survey (N=135 respondents across 15 clinics).

Variables	Univariate statistics, mean (SD)	Aggregation statistics		
		ICC^a^	Rwg(j)^b^	Adm(j)^c^
Implementation climate				
Organizational Readiness for Implementing Change				
Commitment	3.97 (0.25)	0.093	0.84	0.71
Efficacy	3.93 (0.30)	0.044	0.87	0.71
Learning environment				
Internal learning environment	4.86 (0.52)	0.173	0.89	1.01
External learning environment	4.76 (0.63)	0.023	0.74	1.11
Psychological safety	3.75 (0.52)	0.27	0.83	1.02

a Intraclass correlation coefficient; higher values indicate that more variability in responses can be attributed to clinic membership, and thus, more support for aggregation.

b Within-group reliability; higher values indicate greater agreement and more support for aggregation.

c Average deviation index; higher values indicate more dispersion and less support for aggregation

**Table 3.** Baseline clinic level characteristics from the organizational assessment survey (N=135 respondents across 15 clinics).

Clinic Characteristics	Univariate Statistics
Specialty, n (%)	
Rheumatology only	6 (40)
Multispecialty	9 (60)
Ownership, n (%)	
University-owned	12 (80)
Not-university-owned	3 (20)
Size, n (%)	
0-10 clinic members	2 (13)
11-30 clinic members	6 (40)
31 or more clinic members	7 (47)
Number of locations (mean, SD)	2.27 (0.25)
Average clinic years of experience (mean, SD)	2.28 (0.25)
Culture, n (%)	
No dominant culture	11 (73)
Team culture	3 (20)
Rational/Hierarchical culture	1 (7)

#### Clinic Characteristics

The overall response rate for the baseline organizational assessment survey was 93% (n=135), ranging from 30% to 100% across the 15 clinics. Of the study clinics, 6 (40%) were rheumatology-only while the other 9 (60%) were multispecialty (Table 1); 12 (80%) of the study clinics were owned by a university while only 3 (20%) were not owned by a university. Overall, 2 (13%) study clinics had less than 10 members, while nearly half (n=7, 47%) had 31 or more clinic members. Notably, 11 (73%) study clinics had no dominant culture, while 3 (21%) study clinics had a team culture. The average number of years of experience in the clinic was 2.28 (SD 0.25).

On average, clinics in the study reported a relatively high level of organizational readiness to implement the DA, with a mean level of change commitment of 3.97 (SD 0.25) and a mean level of change efficacy of 3.93 (SD 0.30) across the 15 clinics. Similarly, clinics demonstrated high levels of learning climate, with a mean level of internal learning of 4.86 (SD 0.52) and a mean level of external learning of 4.76 (SD 0.63). Clinic personnel’s perception of the permanence of the DA was moderate in the first survey round (6 mo) with a mean of 3.22 (SD 0.74) across all 15 clinics. Univariate analysis also found that perceptions of permanence slightly decreased from the first survey period to the second and third periods (Table 4).

**Table 4.** Descriptive statistics for perceived permanence of the DA at 6-, 12-, and 24-month postimplementation.

Site	Round 1		Round 2		Round 3	
	Total (N)	Mean (SD)	Total (N)	Mean (SD)	Total (N)	Mean (SD)
1	9	3.55 (0.87)	5	3.40 (0.84)	7	3.20 (1.23)
2	7	3.62 (0.49)	4	3.64 (0.71)	6	3.13 (0.24)
3	3	2.33 (0.70)	7	3.24 (1.43)	4	2.65 (0.84)
4	3	2.93 (1.10)	3	2.26 (0.46)	4	1.65 (0.90)
5	7	3.62 (0.73)	3	2.98 (0.52)	5	2.84 (0.59)
6	3	2.86 (0.80)	4	2.90 (1.27)	3	2.20 (1.22)
7	6	3.36 (0.66)	8	3.70 (0.75)	7	3.46 (0.79)
8	4	3.49 (0.34)	6	2.76 (1.16)	2	3.59 (0.84)
9	6	2.70 (0.88)	3	2.73 (1.02)	1	1.60 (0.00)
10	5	3.12 (0.52)	2	3.30 (0.42)	1	1.40 (0.00)
11	11	3.30 90.67)	5	3.40 (0.89)	16	3.84 (0.89)
12	4	2.25 (0.44)	1	3.40 (0.00)	2	2.30 (1.27)
13	1	3.59 (0.00)	2	2.50 (0.70)	1	2.80 (0)
14	5	3.20 (0.20)	4	3.10 (0.20)	8	3.05 (0.65)
15	3	3.46 (0.90)	3	2.93 (0.80)	3	3.87 (0.83)
Total	77	3.22 (0.74)	60	3.13 (0.92)	70	3.12 (0.83)
Cronbach α	0.81		0.90		0.93	
ANOVA	*F* (14,62)=1.86; *P*=.04		*F* (14,45)=0.73; *P*=.72		*F* (14,55)=3.04; *P*=.001	

### Associations Between Organizational Climate Measures and Perceived Permanence of the DA

The multivariate results are reported in Table 5. The internal learning climate was negatively associated with perceived permanence of the DA (β=−1.39; *P*<.05), as was external learning climate (β=−2.11; *P*<.01). Thus, we did not find support for hypotheses 1a and 1b.

**Table 5.** Generalized estimating equation regression results examining the association between organizational climate measures and perceived permanence of the DA, Adjusted for Clinic-level Clustering.

Variables	β (SE)
Independent variables	
Organizational Readiness for Implementing Change	
Commitment	−0.35 (0.73)
Efficacy	4.00 (1.02)^a^
Learning environment	
Internal learning environment	−1.39 (0.56)^b^
External learning environment	−2.11 (0.61)^c^
Psychological safety	2.68 (0.95)^c^
Clinic Characteristic	
Specialty	
Rheumatology only	Reference
Multispecialty	0.38 (0.36)
Ownership	
University-owned	Reference
Not-university-owned	1.04 (0.35)^c^
Size (number of personnel)	
1‐10 clinic members	Reference
11‐30 clinic members	−0.13 (0.30)
31 or more clinic members	0.88 (0.30)^c^
Culture, n, (%)	
No dominant culture	Reference
Team culture	0.73 (0.25)^c^
Respondent covariates	
Education	
High school	Reference
Undergraduate or less	0.04 (0.37)
Professional (eg, MSW and MBA)	−0.31 (0.25)
Doctorate (eg, MD and PharmD)	0.18 (0.26)
Average age (years)	−0.49 (0.53)
Average clinic years of experience	−0.28 (0.07)^a^
Time	
Baseline	Reference
Month	0.05 (0.69)
12-month	−0.19 (0.14)
Total (N)	204

a *P*<.001

b *P*<.05

c *P*<.01

The relationship between the change commitment climate and perceived DA permanence was negative and not statistically significant. However, findings indicated that the change efficacy climate, on average, was positively associated with perceived permanence of the DA (β=4.00; *P*<.001), supporting hypothesis 3.

### Clinic-Level Covariates Included in the Multivariate Models

The strongest and most robust results for control variables were observed for the years of clinic experience, ownership, size, and culture (Table 5). Specifically, the number of years of experience was negatively associated with the perceived permanence of the DA (β=−0.23; *P*<.01). This result suggests that those who have more clinical experience are less likely to continue using the DA. Respondents from nonuniversity clinics reported more positive perceptions of DA permanence than respondents from university-owned clinics (β=1.06; *P*<.01). Finally, relative to members of clinics with no dominant culture, members of clinics with a team culture reported more positive perceptions of DA permanence (β=0.65; *P*<.01).

### Qualitative Results

#### Overview

In total, 36 interviews were conducted across all 15 clinics, with an average of 3.6 (SD 2.6; range 1-8) per clinic. The results below are organized around the major thematic areas of clinic-focused themes and intervention-focused themes.

#### Clinic-Focused Themes

##### Perceived Usefulness of the DA and Ease of Use

One of the key factors likely to support continued use of the DA was a positive attitude toward its usefulness. In general, there was consistent support for the DA from the interviews with clinic members from all sites. The DA was seen as a valuable tool that could simplify the office visit, as the patients potentially could come better informed. Appealing graphics, a user-friendly interface, and straightforward language were highly appreciated. Furthermore, several clinic members provided examples of patients who viewed the DA and became more involved in the decision-making process, asked more questions, or even changed their medication decision, as highlighted by one physician, “It’s a nice way to reinforce some of the most important aspects of how to take care of this disease long term instead of just thinking from visit to visit” (Site 11).

##### Buy-In from Physicians

Despite the perceived usefulness of the DA and alignment with the values of how treatment decisions should be made, a majority of physicians were less inclined to continue using it. One of the physicians noted that he was already adequately educating patients and involving them in decision-making; therefore, he did not have intentions to continue using the DA in the future. This is significant because, according to other nonphysician clinic members, the routine use of the DA would mainly depend on physicians’ interest and judgment. This was also important because patients seemed more open to using the DA when their physician introduced it to them, particularly if they had a good relationship and trusted their physician. Clinic members also noted that if the use of the DA is not promoted, patients may not take the initiative to click on the link and view the DA outside of the office visit. Therefore, the use of the DA in the clinic needed to be endorsed by the physicians if it was to be sustained.

### Differential Utility Depending on the Disease Stage

Another consistent pattern was that, although the DA was regarded by clinic staff as a valuable tool for learning about lupus in general, many felt it would be particularly beneficial for patients who are newly diagnosed. According to several clinic members, patients who had been recently diagnosed with lupus reportedly expressed that they had learned something new from the DA. Given that clinic visits are typically brief, with an average of 15 (SD 5) minutes for return patients and 30 (SD 10) minutes for new patients, physicians feel compelled to be as efficient as possible when conveying information to patients. Therefore, the DA could serve as a valuable source of additional information or a reminder of topics that may have been missed or forgotten during appointments. However, several clinic members noted that, during the study period, the DA was primarily viewed by patients who have had lupus for a long time and did not require medication changes, making it difficult to gage its value. Moreover, because the participating clinics were outpatient, clinic staff believed most patients were not making an active treatment decision. As a result, clinic members observed that many patients, particularly those with longstanding lupus and stable treatment plans, did not perceive the DA as significantly enhancing their knowledge of lupus. However, clinic members suggested that the DA may be more beneficial for patients experiencing disease flares, those undergoing medication changes, or individuals receiving care in inpatient settings where active decision-making is more common.

### Practice Pattern Differences

Clinic members from 5 sites noted that the medication choices presented in the DA did not reflect those used by their clinics for treating lupus. It was recommended to include more scenarios in the DA, as the current version covered either more intense immunosuppressive drugs or those not prescribed by the clinics. Given that the DA often was not applicable to their clinical situations, providers who used different medications inconsistent with the treatment options presented in the DA were unlikely to use it in the future.

### Limited Integration into Routine Clinical Workflow

Almost all clinic members from all 15 sites noted that the DA was seen exclusively as a research tool, with the research coordinator responsible for recruiting and obtaining patient consent, as well as distributing the DA to patients. However, participating in the study imposed certain limitations, as clinics were required to adhere to institutional rules and the DA was not fully integrated into their daily workflow. Most of the time was devoted to research-related tasks, such as identifying eligible patients, obtaining consent, and ensuring that surveys were completed. As a result, the DA was viewed as an “add-on” rather than a clinical tool, making it challenging to garner buy-in from all members of the clinic. Some clinic members noted that if it had been introduced as a clinical tool, it could have been reinforced during weekly physician meetings and better integrated into the clinical workflow.

The DA being viewed only as a research tool had the consequence of limiting involvement from clinic personnel. In many clinics, nurses, medical assistants, and front desk staff were not heavily involved in the study. While they were aware of the study taking place, they did not play a substantial role in distributing the DA. This approach had both advantages and disadvantages. One advantage was that the research coordinator remained the same throughout the study in some clinics, which helped maintain consistency when clinic staff had to leave for various reasons. However, it also limited the potential sustainability of the DA as it was not perceived as a clinical tool that would continue to be used after the study was over.

### Burden of Prescreening/Identifying Patients

Several clinics did not have a dedicated clinic for lupus, so a study coordinator would screen patients beforehand and identify those with lupus based on their *ICD* (*International Classification of Diseases*) code. The coordinator would then seek approval from the physician to include a patient in the study and hand the DA. In other clinics, the physician or principal investigator would identify eligible patients. Regardless of the approach, all clinic members acknowledged the need to identify patients who may be experiencing a flare-up or have recently been diagnosed with lupus in a proactive manner. Ideally, during an office visit, a physician would assess a patient’s eligibility for the DA and inform them that a link would be sent. In addition, the physician should schedule a follow-up visit to answer any questions. However, participants noted that manually prescreening and identifying which patients should view the DA would be a barrier to long-term sustainability.

### Intervention-Focused Themes

#### Customizing the Content and Ensuring Timeliness of the DA

During interviews, clinic members noted that the DA appeared to be more focused on kidney disease and women of childbearing potential. Therefore, it would be important to update and tailor the content to better meet the needs of a diverse group of patients. One frequently cited request was to update the content with new discoveries about medication side effects to increase the likelihood of continued use beyond the study period. Moreover, it would be important to have clearly defined roles/responsibilities so that the DA can be routinely maintained and updated to reflect the latest evidence.

#### Need for Follow-Up if Viewed Outside of Clinic Visit

Although many clinic members preferred the DA to be used independently outside of the consultation, preferably at home, it was hard to guarantee true shared decision-making with this format. The physicians noted that the DA was most effective when it was well-communicated with patients, that is, ensuring that patients are made aware of the availability of the DA, checking patients’ understanding of the information provided, and tailoring the discussion to the patient’s individual needs and preferences. Therefore, even if patients take the time to review the DA at home, it is necessary to have a follow-up call or visit to discuss any issues to really promote shared decision-making. This meaningful discussion would help increase the likelihood of continued use of the DA.

#### Automating/previsit Package

Participants noted that it would be beneficial if the scheduling system could identify patients with lupus and create a patient list to sustain the usage of the DA. Otherwise, turnover in staff makes it difficult to continue using it. Integrating the DA into the patient portal with an alarm for physicians when prescribing a new medication would also be helpful. Automating the process would allow patients to access the DA as a previsit package. Another idea was for physicians to give patients a link to view the DA independently. At the end of the visit, physicians could include the link as part of their instructions for patients to access at home.

## Discussion

### Principal Findings

This study examined the relationship between learning climate, change readiness climate, and the perceived permanence of a lupus DA for patients in 15 geographically diverse rheumatology clinics across the United States. We found that a positive change efficacy climate was significantly associated with the greater perceived permanence of the lupus DA, while change commitment was not significantly associated. Interestingly, both internal and external learning climates were negatively associated with DA permanence. Qualitative findings highlighted challenges to sustainment, including poor workflow integration, lack of physician buy-in, and perceptions of limited applicability of the DA to certain patient populations. Our findings underscore the importance of organizational context for the successful implementation and sustainment of evidence-based interventions, echoing prior theoretical and empirical work [33,34]. Respondents from clinics with a more positive change efficacy climate were more likely to have positive perceptions of DA permanence. This aligns with organizational readiness theory, which posits that efficacy, the belief in collective capability, is a critical driver of change implementation [17].

In contrast, change commitment was not significantly associated with DA permanence. This may be due to the setting of many participating clinics in academic environments with ongoing research and quality improvement projects. Interviews revealed that staff often viewed the DA as a temporary research tool, not a sustained clinical practice. This perception likely undermined broader organizational commitment, despite early engagement. However, the DA being viewed only as a research tool had the consequence of limiting clinic-wide commitment. Most of the clinics had only a handful of isolated research teams designated to implement the study. Thus, the DA did not have a pervasive presence and was rarely articulated as an ongoing explicit priority.

Unexpectedly, more favorable internal and external learning climates were negatively associated with perceived permanence. While this may seem counterintuitive, our qualitative data suggest that supportive learning environments facilitated critical reflection, leading clinic staff to identify limitations of the DA in their specific settings. For example, some physicians realized during implementation that the DA was less applicable to patients with stable disease or those not facing medication changes. Others noted the absence of a reliable method to identify eligible patients, further complicating routine integration. This suggests that learning environments, while essential for innovation adoption, may also lead to early discontinuation if an intervention is perceived as misaligned with clinical needs.

Among the organizational factors, years of experience, culture, and ownership were associated with the perceived permanence of the DA. Interestingly, the results indicated that more experienced members were less optimistic about the likelihood of continuing to use the DA, which is in line with the literature on organizational tenure and its weak positive effect on employee innovative behavior [35]. The interviews shed light on why this might be the case. In most of the clinics, physicians have been with the clinic for 5 or more years, which meant strong institutional knowledge and possibly even a belief they were already adequately educating patients. In addition, as earlier stated, most of the physicians thought that the DA was not quite compatible with their current medication management; thus, they were less likely to continue using the DA. Experiential knowledge of what medications work best for their patients could have substantial influence on their prescription decisions and lead to variations seen in clinical practice [36]. Furthermore, high turnover among support staff meant that physicians were frequently the longest-tenured personnel in these clinics, making them critical for successful sustainment of the DA.

### Practice Implications

The financial and human resources required to develop evidence-based interventions (EBIs) are substantial, making it crucial for practitioners and researchers to learn how to sustain them. This study, based on the implementation of the DA in 15 clinics, highlights the importance of organizational context in sustaining change. The focus should be on enhancing organizational climate, such as readiness for change and learning, before implementation. This study showed that change efficacy had a positive association with perceived DA permanence, while change commitment was not significant. Therefore, early planning that includes all clinic members and discussing the benefits of the EBI can improve buy-in. Likewise, leadership, particularly from physicians, plays a critical role in sustaining innovations in health care settings. A deliberate strategy should be in place to strengthen physician buy-in. Furthermore, if a continued use of the DA requires changes in organizational logistics, organizational management should focus on maintaining flexibility and compatibility of the DA across diverse settings. This includes tailoring the DA’s format to meet the needs of patients and physicians, such as offering options to view it online, through an app, or in the clinic. Finally, a formal strategy for sustaining EBIs should include ongoing evaluation to identify improvement opportunities. Project tracking, reporting, and staff discussions can help address issues early in the implementation process. Regular dissemination of data on patient satisfaction and outcomes, as well as providing incentives for active DA use, can further strengthen the innovation’s presence and sustainability within the organization.

### Limitations

Several limitations should be considered when interpreting this study’s findings. The analysis was based on just 15 clinics, mostly within academic medical centers, limiting the power to detect statistically significant relationships and generalizability. The outcome variable, perceived permanence, may have been biased as data collection occurred during implementation, possibly pressuring participants to respond positively. In addition, limited longitudinal analysis was possible due to staffing turnover, though the qualitative data helped clarify underlying mechanisms behind climate factors from multiple stakeholders’ perspectives.

### Conclusions

Our findings suggest that simply introducing a lupus DA does not guarantee its sustainment in clinical practice. Organizational climate can play a central role in shaping perceptions of an intervention’s permanence. Future research should use longitudinal and multilevel designs to examine how organizational learning, readiness for change, and psychological safety influence the long-term adoption of decision aids. Tracking shifts in perception across time and context can help identify which organizational strategies best support sustained use.

In addition, clearer communication about the DA’s relevance to different disease stages and care pathways is needed. Various versions of the DA were implemented during the COVID-19 pandemic, and confusion about their applicability likely reduced uptake. Future dissemination efforts should ensure that stakeholders understand how different versions of the DA align with specific patient populations. Doing so may improve fit, reduce resistance, and promote sustained integration into routine lupus care.

## Supplementary material

10.2196/69603 Multimedia Appendix 1 Interview Guide for Clinic Personnel.

10.2196/69603 Multimedia Appendix 2 Survey instruments.

## Abbreviations

ADM(J): Average deviation
DA: Decision aid
EBI: Evidence-based interventions
HIPAA: Health Insurance Portability and Accountability Act
*ICD*: *International Classification of Diseases*
IRB: Institutional Review Board
ORIC: Organizational Readiness for Implementing Change
RWG(J): within group reliability
