# Supplementary material for: Organizational Climate and Decision Aid Sustainability in Lupus Care: Mixed Methods Study
Source: JMIR Form Res. 2025 Aug 21;9:e69603. doi: 10.2196/69603 (PMC12369446; doi:10.2196/69603)
Supplement: Multimedia Appendix 1 [file formative-v9-e69603-s001.docx]

Interview Guide for Clinic Personnel

My name is <insert name> and I am here with/on the phone with <insert key informant name>. First, let me thank you for taking time out of your busy schedule to talk with me today. The purpose of today’s interview is to discuss the lupus shared decision-making aid that was implemented in your clinic. In particular, we want to get your views on how effective the decision-aid has been, how effective the implementation strategies were, and the opportunities and challenges to sustaining use of the decision-aid. There are no right or wrong answers, simply your perspectives. These perspectives will help us to make inferences about what clinics are positioned to sustain use of the DA, and why, which can help us understand approaches to working with other clinics to implement the DA in ways that are sustainable. Before getting into the specific questions, let’s first take care of a couple of important issues.

Today’s interview will take approximately 45 to 60 minutes. With your permission, today’s interview will be recorded. The information that we gather is confidential and no one else outside of the evaluation team will access the specific information you provide. It is our intention, however, to summarize the information provided by you and other people we interview from the clinic in a report back to clinic leadership for the purposes of identifying the most appropriate implementation strategies for your clinic.

Your participation in today’s interview is entirely voluntary. Whether you choose to participate or not will have no bearing on your job or any work-related evaluations. You may also change your mind later and stop participating at any time, even if you agreed to participate earlier. Likewise, if you do not want to answer any of the questions during the interview, you can say so and I will move on to the next question.

So, before we begin:

Do I have your consent to participate in today’s interview?

1. If yes, do I have your permission to record the interview?
2. If no, “thank you for your time”.

**Background**

First, I’d like to get a little background information about you.

1. Tell me about your role in the organization.
2. How long have you worked in the clinic?
3. What is your educational and professional background?

**Effectiveness**

Next, I’d like to talk about the effectiveness of the DA.

1. How effective do you think the lupus shared decision-aid has been in your clinic?
2. In your opinion, what are the strengths of the decision-aid? Weaknesses?
3. What could be done to improve the effectiveness of the decision-aid in your clinic?
4. What types of adaptations, if any, were made to the decision aid or the implementation of the decision aid in the clinic over the past 2 years?
   1. Why were these adaptations necessary?
   2. How did they ‘improve’ the decision aid and/or its fit with your organization?
5. Did your clinic experience any turnover in key personnel involved with the decision aid over the past 2 years?
   1. If yes, how did that affect the use of the decision aid
      1. Probe: did turnover affect how staff implemented the decision aid
   2. If no, how important do you think workforce stability is for effective use of the DA? Why is that?

**Implementation Strategies**

Let’s turn now to the implementation strategies used by the clinic. Your clinic used <list strategies used>.

1. How effective was the overall implementation strategy?
2. Were some components more effective than others?
   1. Which ones and why?
   2. For those components that you thought were less effective, why do you think they were less effective?
      1. Probe: resources/infrastructure? Poor fit with organizational culture or goals?
3. Do any of the components work in complementary or synergistic fashion with each other?
4. Similar question, do think any of the components work at conflicting or cross- purposes?
5. Reflecting back on the overall implementation experience, what things would you have done differently, if anything

**Sustainability**

The final set of questions pertains to sustainability or the continued use of the DA in the future.

1. Knowing what you know now about the decision aid, how well do you think the decision fits with the clinic’s values, goals, structure, resources, and patient base?
2. In your opinion, how likely is it that the clinic will continue to use the DA?
   1. If not likely, why not?
   2. If yes, what types of adaptations do you feel will be necessary to continue to use the DA?
3. In your opinion, what types of things would help your clinic continue to use the decision aid in the future?
4. How could clinic leaders support efforts to continue to use the decision aid?
5. To what degree has the decision aid been integrated into work processes?
   1. Can you provide an example where the decision has been integrated into existing work processes or where work processes were modified to accommodate the decision aid?
