# Supplementary material for: Organizational Climate and Decision Aid Sustainability in Lupus Care: Mixed Methods Study
Source: JMIR Form Res. 2025 Aug 21;9:e69603. doi: 10.2196/69603 (PMC12369446; doi:10.2196/69603)
Supplement: Multimedia Appendix 2 [file formative-v9-e69603-s002.docx]

Perceived DA Permanence Survey

| 1 | 2 | 3 | 4 | 5 |
| --- | --- | --- | --- | --- |
| Disagree | Somewhat Disagree | Neither Agree nor Disagree | Somewhat Agree | Agree |

| Our clinic has changed organizational procedures to sustain use of the decision aid. | 1 | 2 | 3 | 4 | 5 |
| --- | --- | --- | --- | --- | --- |
| Our clinic has changed clinical procedures to sustain use of the decision aid. | 1 | 2 | 3 | 4 | 5 |
| Our clinic has dedicated financial resources to sustain use of the decision aid. | 1 | 2 | 3 | 4 | 5 |
| Our clinic has dedicated staff resources to sustain use of the decision  aid. | 1 | 2 | 3 | 4 | 5 |
| The decision aid is a permanent part of our clinic. | 1 | 2 | 3 | 4 | 5 |


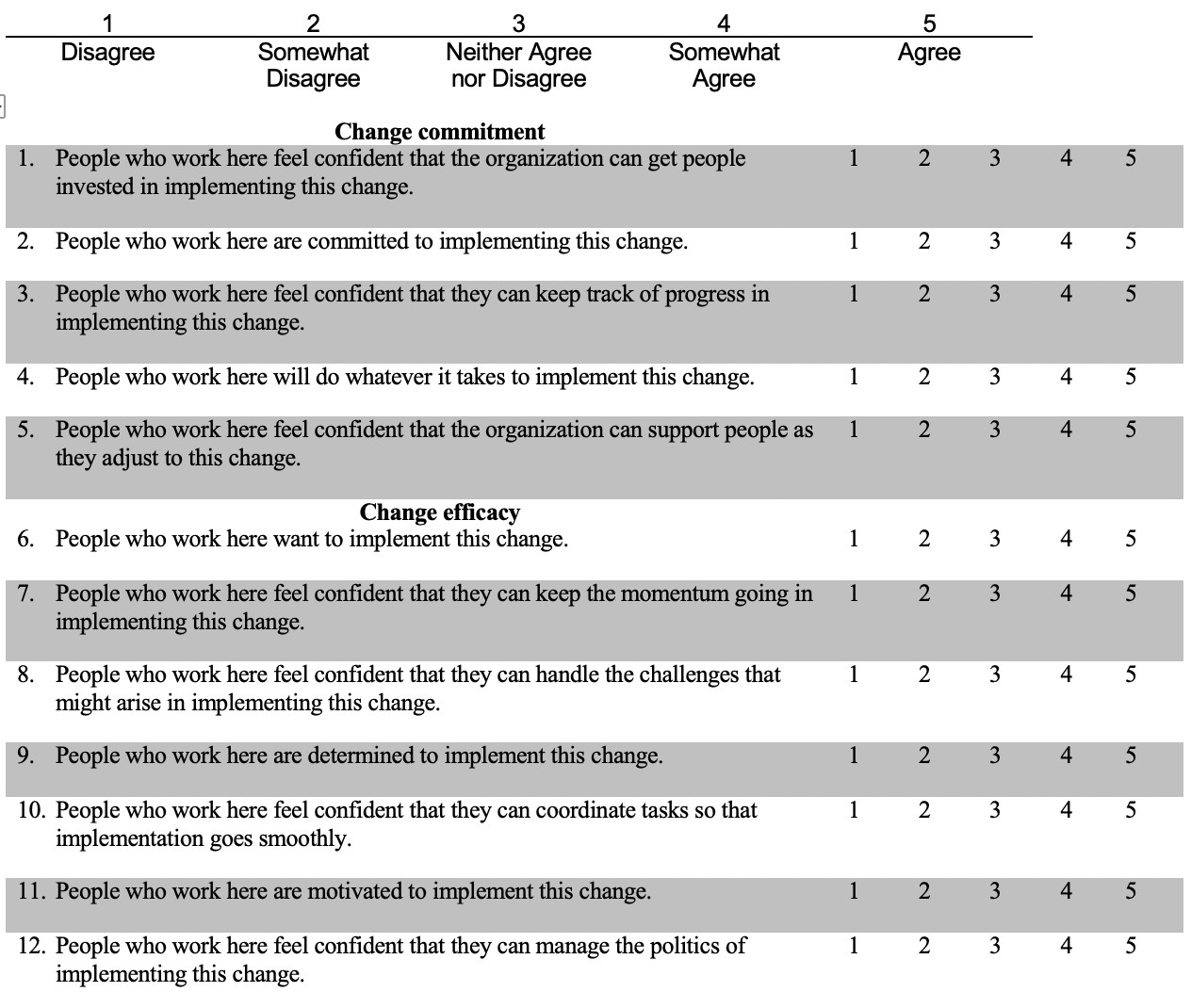
Organizational Readiness for Change

Organizational Learning

| 1 | 2 | 3 | 4 | 5 | | 6 | | | | 7 | | | |  |  |
| --- | --- | --- | --- | --- | --- | --- | --- | --- | --- | --- | --- | --- | --- | --- | --- |
| Strongly disagree | Disagree | Somewhat disagree | Neither agree nor disagree | Somewhat agree | | Agree | | | | Strongly agree | | | |  |  |
| **Internal learning** | | | | | | | | | | | | | |  |  |
| 1. Problems and errors in this clinic are always communicated to the appropriate people for action can be taken | | | | | | 1 | | 2 | 3 | 4 | | 5 | 6 | 7 | |
| 2. We often take time to figure out ways to improve our clinic's work processes. | | | | | | 1 | | 2 | 3 | 4 | | 5 | 6 | 7 | |
| 3. In this clinic, people talk about mistakes and suggest ways to prevent and learn from them. | | | | | | 1 | | 2 | 3 | 4 | | 5 | 6 | 7 | |
| 4. This clinic tend to handle conflicts, and differences privately or offline, as opposed to addressing them directly as a group. | | | | | | 1 | | 2 | 3 | 4 | | 5 | 6 | 7 | |
| 5. This clinic frequently obtains new information that leads us to make important changes in our plans or work processes. | | | | | | 1 | | 2 | 3 | 4 | | 5 | 6 | 7 | |
| 6. Members of this clinic often raise concerns that they have about clinic plans or decisions. | | | | | | 1 | | 2 | 3 | 4 | | 5 | 6 | 7 | |
| 7. This team constantly encounters unexpected hurdles and gets stuck. | | | | | | 1 | | 2 | 3 | 4 | | 5 | 6 | 7 | |
| 8. We try to discover assumptions or basic beliefs about issues under discussion. | | | | | | 1 | | 2 | 3 | 4 | | 5 | 6 | 7 | |
| 9. This team is not very good at informing key people of the need to buy into what the team is planning and accomplishing. | | | | | | 1 | | 2 | 3 | 4 | | 5 | 6 | 7 | |

**External learning**

10. We don't have time to communicate information about our clinic's work to others outside the clinic.

1 2

3 4 5 6 7

11. We don't have time to communicate information about our clinic's work to

others outside the clinic.

1 2 3 4 5 6 7

12. We invited people from outside of the clinic to present information or have discussions with us.

1 2

3 4 5 6 7

13. This clinic retrieves all of the information possible from several different

sources.
